# Development of a Sequential Fractionation-and-Recovery Method for Multiple Anti-Inflammatory Components Contained in the Dried Red Alga Dulse (*Palmaria palmata*)

**DOI:** 10.3390/md21050276

**Published:** 2023-04-28

**Authors:** Ga-Hyun Joe, Masafumi Masuoka, Ryosuke Reisen, Seiya Tanaka, Hiroki Saeki

**Affiliations:** 1 Faculty of Fisheries Sciences, Hokkaido University, Hakodate 041-8611, Japan; 2 Department of Food Science and Technology, Tokyo University of Marine Science and Technology, Tokyo 108-8477, Japan

**Keywords:** red algae, dulse, anti-inflammatory component, separation, extraction, phycobiliprotein, chlorophyll

## Abstract

A separation process was established to sequentially fractionate and recover three anti-inflammatory components derived from sugars, phycobiliprotein, and chlorophyll from the hot-air-dried thalli of the red alga dulse (*Palmaria palmata*). The developed process consisted of three steps, without the use of organic solvents. In Step I, the sugars were separated by disrupting the cell wall of the dried thalli with a polysaccharide-degrading enzyme, and a sugar-rich extract (E1) was obtained by precipitating the other components, which were simultaneously eluted by acid precipitation. In Step II, the residue suspension from Step I was digested with thermolysin to obtain phycobiliprotein-derived peptides (PPs), and a PP-rich extract (E2) was obtained by separating the other extracts using acid precipitation. In Step III, solubilized chlorophyll was obtained by heating the residue, which was acid-precipitated, neutralized, and re-dissolved to concentrate the chlorophyll-related components (Chls)-rich extract (E3). These three extracts suppressed inflammatory-cytokine secretion by lipopolysaccharide (LPS)-stimulated macrophages, confirming that the sequential procedure had no negative effects on the activities of any of the extracts. The E1, E2, and E3 were rich in sugars, PPs, and Chls, respectively, indicating that the anti-inflammatory components were effectively fractionated and recovered through the separation protocol.

## 1. Introduction

Recently, it has become clear that continuous mild inflammation and its exacerbation in humans, which are attributable to unhealthy lifestyles, encourage the development of various non-communicable diseases, such as cancer, diabetes, and cardiovascular and respiratory diseases [1,2]. From the viewpoint of maintaining health there is increasing interest in improving inflammatory status by ingesting food groups with anti-inflammatory activities [3,4]. The exploration of food materials with anti-inflammatory activities and the development of techniques for their utilization are important research topics, in a similar manner to the utilization studies on anti-oxidant and anti-cancer ingredients. Research activities of this type are underway for various foods, such as edible plants [5], dairy products [6], and seafoods [7].

Macroalgae, also called seaweeds, are highly productive marine biomasses that contain a wide variety of bioactive components. For example, algae, including dulse, the material used in this study, are important sources of iodine, an essential trace element for humans [8,9], and various health benefits of these elements, such as immunomodulatory, antitumor, antioxidant, and anti-bacterial activities, have been elucidated through in vitro and in vivo studies [10,11,12,13]. These findings indicate that algae have significant potential as anti-inflammatory food materials that could greatly contribute to the maintenance of human health, and many researchers are attempting to use algal components as functional food ingredients [14,15].

Algae, which generally have a moisture content of 95% or higher, are often hot-air-dried to reduce their volume when used as raw materials for food or bioindustrial applications [16,17], thereby improving their storage and transportability. However, the drying process induces the hardening of the thallus and the thermal denaturation of the functional components, resulting in a loss of extractability in various functional components [18]. Various extraction methods for bioactive components have been developed, such as ultrasound, microwave, pressurized liquid, osmotic shock, and pulsed electric field [19,20,21,22]. In particular, the enzymatic decomposition of cell walls is effective for extracting bioactive components from raw algal thalli [23,24] without the use of specialized equipment. However, few studies have examined the efficient extraction of compounds from dried and hardened thalli. Polysaccharides, which are relatively thermostable, are more easily extracted from dried thalli by disrupting hardened cell walls, whereas the extractability of phycobiliproteins and chlorophylls is greatly reduced [18] by the hot-air-drying process. Therefore, it is important to develop a separation technology to recover these functional components from dry thalli without impairing their efficacy, since such a technology would greatly contribute to the effective use of algae as functional materials to improve human health. 

This study aimed to establish a novel separation protocol for the sequential fractionating and recovery of three anti-inflammatory components with different chemical properties (sugars, phycobiliprotein-derived peptides (PPs), and chlorophyll-related components (Chls)) in dried red algae. Dulse (*Palmaria palmata*), a popular edible seaweed found at high latitudes in the northern hemisphere [25], which is rich in phycobiliproteins, with various biological functions [26,27], was selected as the research material in this study. Conclusively, in this study, we succeeded in recovering anti-inflammatory components from dried dulse thalli without using organic solvents or causing a loss of function. The key factors in the developed scheme were enzymatic-cell-wall weakening, the enzymatic degradation of phycobiliproteins, and the removal of the phytol group from the chlorophylls. Moreover, the anti-inflammatory properties of the three obtained fractions were compared by assessing their effects on LPS-stimulated mouse macrophages.

## 2. Results and Discussion

### 2.1. Sequential Procedure for Fractionating Anti-Inflammatory Components in Dulse

**Figure 1:**
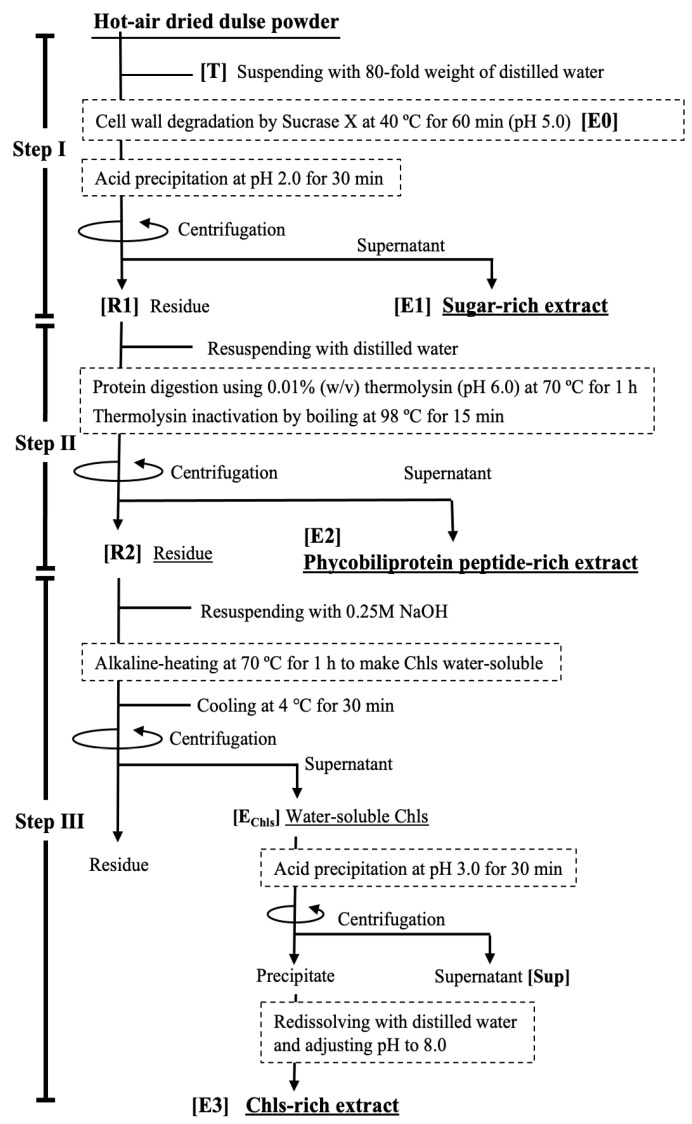
Outline of a continuous separation process for recovering multiple anti-inflammatory components from dulse.

Three fractions enriched in anti-inflammatory components, namely digested sugars, PPs, and Chls, were obtained via the three-step sequential fractionation procedure. The outline of the sequential fractionation-and-recovery method is shown in Figure 1. The separated components recovered were E1 in Step I, E2 in Step II, and E3 in Step III. In this study, the behavior of the three components in each separation step was investigated to establish the effective fractionation conditions.

#### 2.1.1. Selective Extraction of Sugars from Dried Thalli by Combined Enzymatic Degradation and Acid Precipitation (Step I)

**Figure 2:**
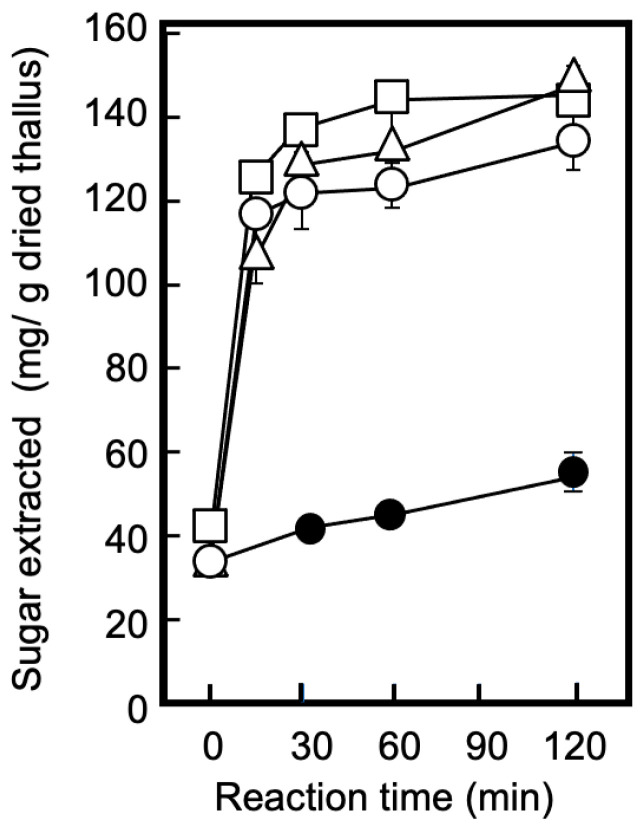
The effect of Sucrase-X treatment on sugar extraction from dried thalli. Sucrase X (polysaccharide-degrading enzyme containing xylanase as the main component) at different final concentrations (*w/v*) was added to dulse-dried thallus suspension (pH 5.0) and reacted at 40 °C for 0–120 min. The amount of extracted sugar in E0 (Figure 1) was examined. Enzyme concentrations: 0% (closed circle), 0.001% (open circle), 0.0025% (open triangle), and 0.005%% (open square).

Cell-wall degradation using polysaccharide-degrading enzymes is a useful step for extracting bioactive compounds [28,29], and it was applied to the dried hardened thalli in this study. In Step I of the separation outline, illustrated in Figure 1, dried thalli suspended in distilled water were treated with Sucrase X to effectively extract the sugars, followed by acid precipitation to completely retain phycobiliproteins and chlorophylls in the precipitate. Figure 2 shows the effect of the enzyme concentration and reaction time on the amount of sugars extracted for E0. When the thallus suspension (pH 5.0) was continuously stirred, the amount of extracted sugars increased slightly with the passage of time, reaching 49.0 ± 6.6 mg/g dried thallus after 120 min. Conversely, the thallus suspension containing Sucrase X exhibited a rapid increase in the amount of extracted sugars and plateaued at approximately 150 mg/g of dried thallus after 60 min, regardless of the enzyme concentration (0.001–0.005%). The increase in the amount of extracted sugars was clearly attributable to cell-wall degradation. As shown by the results in Figure 2, the conditions for the Sucrase-X treatment in Step I were set to an enzyme concentration of 0.0025%, a temperature of 40 °C, and an exposure time of 60 min, considering the optimal pH and temperature of Sucrase X.

**Figure 3:**
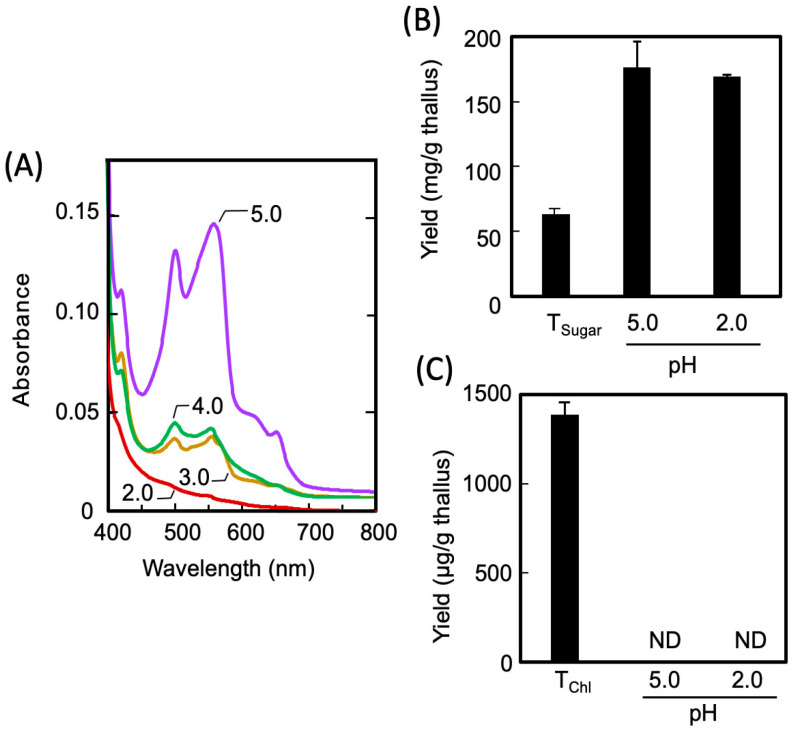
The effects of pH on the solubility of sugars, phycobiliproteins, and Chls in E0. The effects of pH on the solubility of phycobiliproteins (**A**) sugars (**B**), and Chls (**C**) in extracts (E0 in Figure 1) were examined. Numbers in (**A**) indicate the pH of the extracts. The T_sugar_ in (**B**) is the amount of extracted sugar (pH 5.0) without Sucrase-X treatment. Chlorophyll measurements in (**C**) were performed under 80% acetone. The T_Chol_ is the amount of extracted Chls from dried thalli.

The Sucrase-X-treated extract (E0 in Figure 1) obtained from the dried thalli had a transparent red color; the absorption spectrum of the E0 shown in (Figure 3A, solid line) indicates the presence of the extracted phycobiliprotein in the E0 (phycocyanin: 495 nm and 515 nm; phycoerythrin: 610 nm). Thus, to completely separate and recover the phycobiliproteins from the E0, the phycobiliproteins were acid-precipitated to decrease the pH of the extract to 2–4. As a result, the phycobiliprotein content in the E0 diminished as the pH decreased, and the protein was completely transferred to the extracted residue (R1) in Step 1 through acid treatment at pH 2.0, as presented in Figure 3A. As shown in Figure 3B, the acid treatment had no effect on the solubility of the sugars in the E0. Furthermore, as shown in Figure 3C, no chlorophylls were eluted from the thalli, regardless of the Sucrase-X or acid treatment. The results in Figure 2 and Figure 3 demonstrate that in Step I, enzymatically degraded sugars were extracted while both the phycobiliproteins and the chlorophylls were retained in the R1. The resulting sugar-rich extract was denoted as E1, as presented in Figure 1.

Xylan, the main component of dulse, contains mixed β-(1→3)/β-(1→4)-xylan bonds [30], which are not found in land plants. Therefore, the sugar fractions recovered from dulse using organic solvents and polysaccharide-degrading enzymes tend to contain large amounts of xylo-oligosaccharides. with a high degree of polymerization [31]. In this experiment, we found xylo-oligosaccharides with a degree of polymerization of 2–4 in the E1 using the LC/MS/MS analysis [32], as shown in Figure S1 (Supplementary Data). Further research is needed on the composition of E1 and the detection of the presence of functional xylo-oligosaccharides with higher degrees of polymerization [33].

#### 2.1.2. Recovery of Phycobiliprotein-Derived Digested Peptides from the Thallus Residue (Step II)

**Figure 4:**
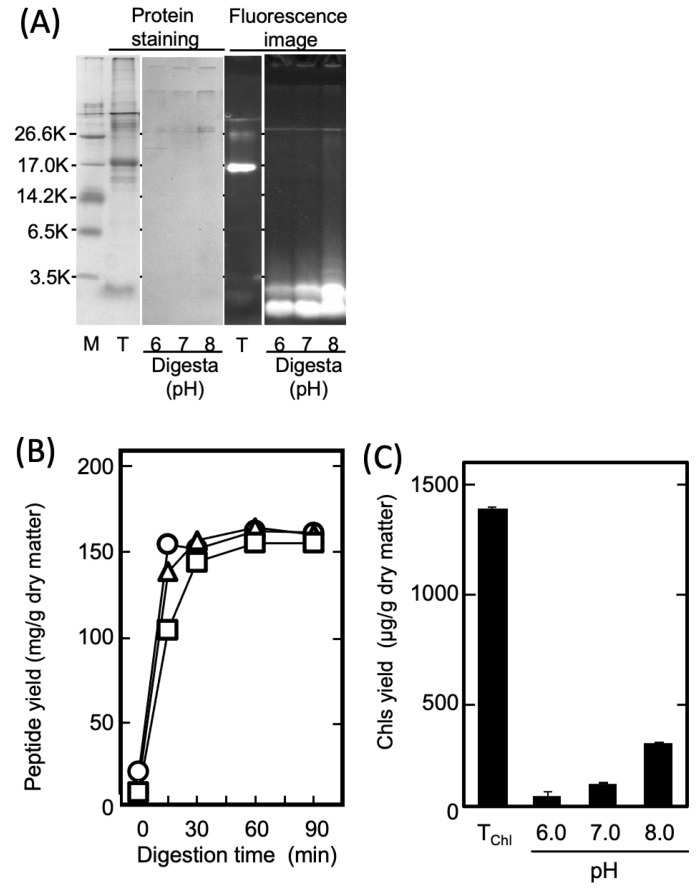
The effect of pH on the selective extraction of PPs by thermolysin digestion. (**A**) SDS-PAGE patterns of PPs in E2 produced by thermolysin digestion at pH 6.0–8.0. M: molecular-weight marker. T: whole extract from dried thalli shown in Figure 1. “Digesta” indicates PPs produced at pH 6.0, 7.0, and 8.0. (**B**) PPs produced by thermolysin digestion at pH 6 (open circle), 7 (open triangle), and 8 (open square). (**C**): Eluted Chls following digestion of R1 by thermolysin at pH 6.0–8.0. The Chl yield was measured under 80% acetone. The T_Chl_ shows the amount of Chls extracted from dried thalli by 80% acetone.

As shown in Figure 3A, the phycobiliproteins contained in the dried thalli were mostly retained in the precipitated residue (R1) in Step I because they lost solubility following acid denaturation. However, it has been reported that PPs exhibit anti-inflammatory activities [27]. Therefore, in Step II, the anti-inflammatory peptides (PPs) were prepared by treating the phycobiliproteins retained in R1 with protease. To prepare the PP-rich fraction, E2, the R1 was suspended in distilled water, adjusted to 6.0, 7.0, or 8.0 (the optimal pH range of thermolysin) [34], and treated with 0.01% (*w/v*) thermolysin at 70 °C for 90 min. Figure 4A shows the SDS-PAGE pattern of the PPs in the E2 prepared under each condition. Evidently, the fluorescent phycobiliproteins were degraded to peptides, with molecular weights of less than 3500, irrespective of the digestion pH. Additionally, Figure 4B shows the time dependence regarding the amount of eluted water-soluble peptides during the thermolysin digestion. The yield of the PPs obtained through the thermolysin digestion nearly peaked after 30 min at all the pH levels. Conversely, as shown in Figure 4C, the amount of Chls that co-eluted with the digested peptides decreased in a pH-dependent manner and reached its nadir at pH 6. The results in Figure 4C demonstrate that the pH adjustment in the enzymatic digestion was important for the selective preparation of the PPs in Step II, and that the thermolysin digestion at pH 6.0 was effective for preparing the PP-rich fraction, E2.

#### 2.1.3. Recovery of Chls from the Thallus Residue after Removing Sugars and Phycobiliproteins (Step III)

**Figure 5:**
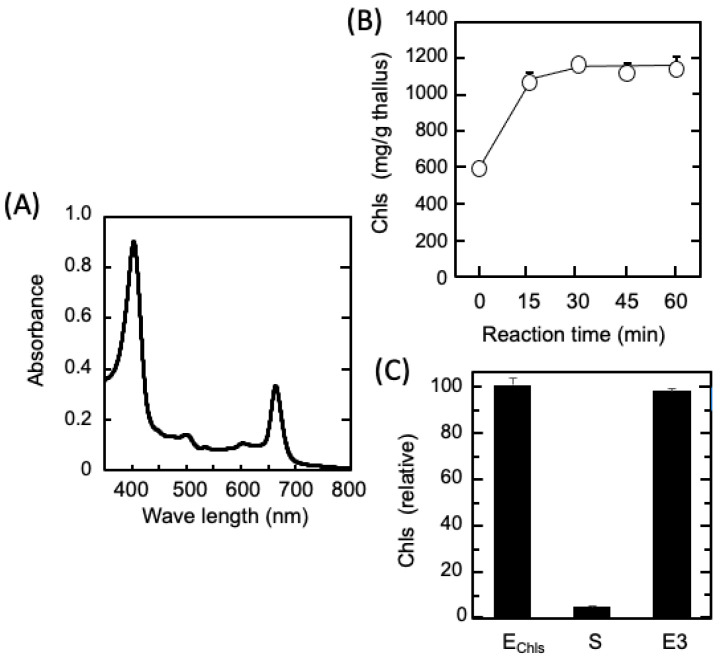
Recovery and concentration of Chls collected from R2 and R3. (**A**) Absorption spectrum of E_Chls_ extracted from R2 by alkaline heat treatment. (**B**) Effect of the alkaline-heat-treatment time on the extraction of Chls from R2. (**C**) Recovery and resolubilization of water-soluble Chls by acid precipitation. E_Chls_: Alkaline-heat-treated Chl extract. S: Chls remaining in the centrifugal supernatant after acid precipitation. E3: the Chl-rich extract shown in Figure 1. Their relationship is explained in Figure 1.

In Step III, the Chls remaining in the R2 were extracted and recovered without using an organic solvent. That is, the R2 was resuspended in 250 mM NaOH (pH 13), solubilized by heating at 70 °C for 1 h, immediately cooled, and centrifuged to obtain the extract, which was transparent green in appearance. As shown in Figure 5A, its absorption spectrum exhibited two peaks (405 and 663 nm) characteristic of the tetrapyrrole ring, and the absorption maximum in 80% ethanol was the same as that of the chlorophyll a extracted from the dried thalli. Because the properties were consistent with those of chlorophyllide, a water-soluble analog of chlorophyll [35], it is clear that the phytol groups of the chlorophyll were removed by the alkaline heat treatment, resulting in Chls composed mainly of water-soluble chlorophyllide. In Figure 5B, which presents the results of the Chl extraction from the R2 over time, the amount of extracted Chls nearly peaked after 30 min of alkaline heat treatment. 

Subsequently, as shown in Figure 5C, when the pH of the extract was lowered to 3.0, almost all the Chls were insolubilized and removed from the supernatant, and 97% of the Chls were concentrated in the precipitate. Finally, all of the acid-precipitated Chls were collected into the Chl-rich extract, E3, through resuspension in water and re-dissolution at pH 8.0, without using an organic solvent.

### 2.2. Composition of Each Fraction and Their Anti-Inflammatory Effects

Table 1 shows the distribution ratio of the sugars, PPs, and Chls at each preparation step, which was calculated from the data in Figure 3, Figure 4 and Figure 5. The distribution ratios of the sugars, PPs, and Chls were 91.7:7.6:0.7 in E1, 9.8:80.9:9.3 in E2, and 0.9:3.6:95.5 in E3. These results indicated that the anti-inflammatory components were effectively fractionated and recovered in Steps I–III, presented in Figure 1. By applying common fractionation techniques, it is not difficult to improve the purity of the main components of each extract. For example, electrolytes, such as amino acids contaminating E1, can be easily removed by ion-exchange chromatography. In addition, the repeated acid precipitation of E3 effectively removes mixed oligosaccharides.

**Figure 6:**
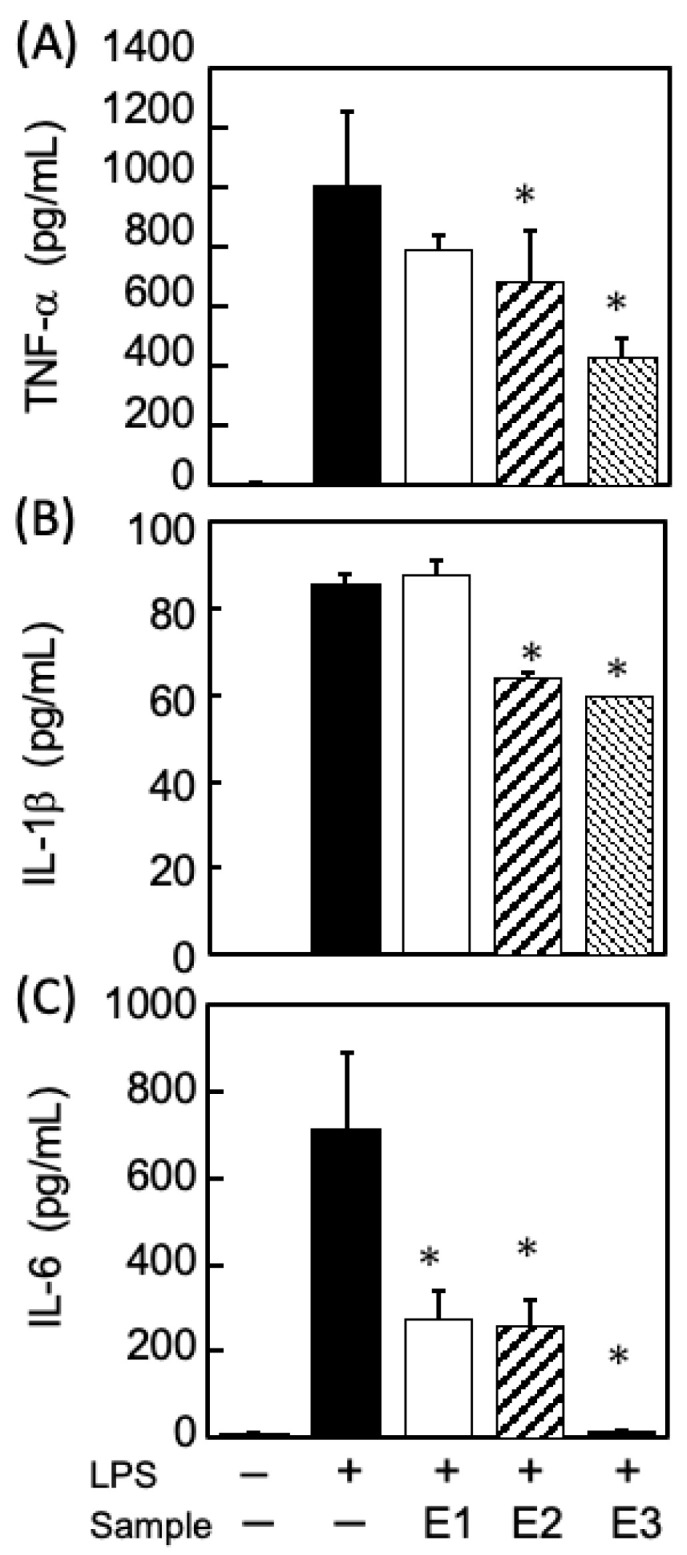
Comparison of the suppressive effects of each extract on the secretion of inflammatory cytokines by LPS-stimulated macrophages. (**A**) TNF-, (**B**) IL-1, and (**C**) IL-6. * *p* < 0.05 vs. LPS-stimulated macrophages (black bar), Dunnett’s test.

In this study, it was necessary to ensure that the three fractions demonstrated comparable anti-inflammatory activities to the dulse extract prior to fractionation. Therefore, the anti-inflammatory potential of the E1, E2, and E3 fractions was evaluated using lipopolysaccharide (LPS)-stimulated mouse macrophages (RAW-264.7 cells). As presented in Figure 6, the E2 and E3 simultaneously suppressed the secretion of TNF-a, IL-1b, and IL-6. These results were consistent with the previously reported inhibitory effects of PPs and Chls, respectively, on the secretion of inflammatory cytokines [27]. Given that both the E2 and the E3 effectively suppressed the secretion of all three cytokines in the LPS-stimulated macrophages, they may have exerted inhibitory effects on the signaling pathways associated with the LPS receptor, TLR4, which is important for triggering inflammation [36,37].

In Figure 6, the differences between the inhibitory effects among the three fractions was most prominently observed in the IL-6 secretion, which was markedly suppressed by E3 (Chls-rich fraction). Furthermore, the E1, which contains enzymatically degraded xylose products, only suppressed the secretion of IL-6. Indeed, several studies [38,39,40] reported that xylose and xylo-oligosaccharides extracted from algae strongly suppressed IL-6 secretion among the cytokines produced in LPS-stimulated macrophages, compared to TNF-alpha and IL-1B.

It is known that the TLR4-MAPK/NF-κB pathway is the major signaling pathway involved in LPS-stimulating inflammation [36,37]. However, E1 may have a different anti-inflammatory mechanism from those of the other two fractions, as it may be involved in specific signaling pathways that induce IL-6 secretion in macrophages. These distinct characteristics of the three anti-inflammatory fractions may enable a wide range of applications of dulse as an anti-inflammatory agent. In the future, we plan to investigate the mechanism of action for each anti-inflammatory fraction, focusing on the analysis of signaling pathways and their effects on macrophage differentiation, which is closely related to anti-inflammation.

## 3. Materials and Methods

### 3.1. Materials

Fresh dulse was washed with water, stored at −25 °C for 1 week, and then dried in hot air at 50 °C and 10% relative humidity for 16 h using a constant-temperature and -humidity cabinet (Taiyo Seisakusho Co., Ltd., Hokuto, Hokkaido, Japan). The dried thalli were pulverized using an electric mill, and were subsequently passed through a 250-µm test sieve (Japan Industrial Standard Z8801 for Test sieves of metal wire cloth). The resulting hot-air-dried dulse powder was sealed, together with silica gel, and stored at 4 °C until use. The RAW 264.7 murine macrophages were supplied by American Type Culture Collections via Dainippon Sumitomo Pharma Co., Ltd (Osaka, Japan).

### 3.2. Chemicals

Sucrase X™, an edible glycoside hydrolase agent containing xylanase as its main ingredient, was purchased from Mitsubishi Chemical Cooperation (Tokyo, Japan). Thermolysin (EC 3.4.24.27, from *Bacillus thermoproteolyticus rokko*) was supplied by Wako Pure Chemical Industries (Osaka, Japan). Cell-culture reagents were purchased from Life Technologies (Carlsbad, CA, USA). The LPS (from *E. coli* O111:B4) was obtained from Sigma-Aldrich (St. Louis, MO, USA). Dulbecco’s modified Eagle’s medium (DMEM), fetal bovine serum (FBS), and penicillin–streptomycin were purchased from Thermo Fisher Scientific (Waltham, MA, USA). Non-essential-amino-acid solution was purchased from Nacalai Tesque (Kyoto, Japan). Adhesive culture flasks (bottom area, 25 cm²) and 96-well cell-culture plates were purchased from Corning (Corning, New York, NY, USA). All other chemicals used in the study were purchased from Kanto Chemical Co., Inc. (Tokyo, Japan).

### 3.3. Fractionation and Recovery Method for Multiple Anti-Inflammatory Components

Figure 1 shows the scheme for separation and recovery of three anti-inflammatory-component-rich extracts from dried dulse powder, namely sugars, PPs, and Chls. These extracted fractions were separated in three steps: E1, E2, and E3, respectively. In Step I, the dried thallus powder was suspended in an 80-fold volume of distilled water, and the pH was adjusted to 5.0 by 6 M HCl, followed by the addition of Sucrase X at a final concentration of 0.0025% (*w/v*). After heating at 40 °C for 60 min to degrade the cell wall, the pH of the suspension (E0) was adjusted to 2.0, followed by incubation for 30 min and centrifugation. The supernatant was fractionated as the sugar-rich extract (E1). In Step II, R1 was resuspended in the same volume of distilled water used in Step I, the pH was adjusted to 6.0, and then 0.01% (*w/v*) thermolysin was added. After proteolytic digestion at 70 °C for 1 h and then boiling for 15 min to inactivate thermolysin, the digesta was cooled and centrifuged. The resulting supernatant was collected as the PP-rich extract (E2). In Step III, R2 was resuspended in 0.25 M NaOH for 30 min, followed by heating at 70 °C for 1 h to remove the phytol group from chlorophyll to make it water-soluble. After cooling at room temperature, the alkaline-heated extract containing Chls was collected by the same centrifugal condition. The pH of this extract was then adjusted to 3.0 using 6 M HCl, the mixture was stirred for 30 min, and Chls were collected in the precipitate. Finally, the precipitate was mixed with distilled water, adjusted to pH 8.0, and dissolved for 60 min to obtain the Chls-rich extract (E3). All preparation processes were performed at 4–8 °C, and centrifugation was performed at 20,000× *g* for 30 min. 

### 3.4. Quantitative Analysis of the Components Extracted from Dried Thallus

The amount of the extracted components was expressed as the yield from 1.0 g of each dried thallus. Sugar content was measured by the phenol–sulfuric-acid method [41], and xylose was used as the standard sugar. The PP content was determined by the Lowery method [42] using bovine serum albumin fraction V (Sigma-Aldrich) as the standard. The Chls were dissolved in acetone at a final concentration of 80%, sealed, stirred at 25 °C for 30 min, and subjected to an absorption-spectrum assay. The concentration, as equivalents to chlorophyll a in µg/mL, was calculated using the following equation: 12.25 × A_663.6_ − 2.55 × A_646.6_, where A_663.6_ and A_646.6_ were the absorbance at 663.6 and 646.6 nm, respectively, in 1 cm of the optical path length [43]. 

### 3.5. Sodium Dodecyl Sulfate–Polyacrylamide Gel Electrophoresis (SDS-PAGE)

The protein contents of the extract were subjected to SDS-PAGE [44], followed by protein staining using Bio-Safe CBB G-250 Stain (Bio-Rad, Richmond, CA, USA) and fluorescence imaging using a gel-documentation system equipped with a light-emitting diode (LED) illuminator (peak wavelength of LED for excitation: 525 nm; Light Capture II/VISRAYS-GL, ATTO, Tokyo, Japan). Each assay sample was dissolved in an equal volume of 2% SDS–20 mM Tris–HCl (pH 8.0) containing 8 M urea and 2% 2-mercaptoethanol, and then heated in boiling water for 2 min. Meanwhile, 12.5% acrylamide gel was used, and equal volumes of the SDS-treated samples were loaded into each gel lane. A protein-marker kit (Sigma-Aldrich) was used to estimate the molecular masses of the extracted protein subunits.

### 3.6. Evaluation of the Anti-Inflammatory Activity of the Extracts Using Mouse Macrophages

Each extract (E1, E2, and E3) was evaluated for its anti-inflammatory activity by investigating the suppression of pro-inflammatory-cytokine production by LPS-stimulated mouse macrophages. The RAW 264.7 cells were cultured in DMEM containing 10% heat-inactivated FBS, 10,000 units/mL penicillin, 10,000 μg/mL streptomycin, and 0.1 mmol/L non-essential amino acids at 37 °C in a 5% CO_2_ humidified atmosphere. In TNF-α and IL-6 measurements, RAW 264.7 cells in DMEM containing 10% FBS were seeded into 96-well plates at 3.0 × 10⁴ cells/well and cultured for 2 h, and then the medium was replaced with DMEM containing 0.1% FBS. 

Prior to the cell experiments, the cytotoxicity of the extracts was examined in RAW 264.7 cells, and the CCK-8 assay (Dojindo, Kumamoto, Japan) was used to measure the effect of the extracts on cell viability [45]. The E1 and E2 did not affect the viability of macrophages, whereas E3 at concentrations of 100 μg/mL decreased cell viability (data not shown). Therefore, the E3 fraction was used at 50 μg/mL. Based on the results of the preliminary experiments, after serum starvation for 24 h, the cells were treated with E1 (500 µg/mL), E2 (500 µg/mL), or E3 (50 µg/mL) for 24 h under stimulation with 20 ng/mL LPS. To analyze IL-1β production, RAW 264.7 cells were seeded in 96-well plates at a density 5.0 × 10⁴ cells/well, serum-starved overnight, and treated with E1 (500 μg/mL), E2 (500 μg/mL), or E3 (50 μg/mL) for 72 h under LPS stimulation at 20 μg/mL. After finishing treatment with each extract (E1–E3), the cell supernatant was collected by centrifugation, and TNF-α, IL-1β, and IL-6 levels in the cell medium were quantified using enzyme-linked immunosorbent assay kits (BioLegend, San Diego, CA, USA), according to the manufacturer’s protocol.

### 3.7. Statistical Analysis

Statistical analysis was performed using JMP software (version 13.0; SAS Institute Japan Inc., Tokyo, Japan). All values are presented as the mean and standard error of the mean. Comparisons in each group were performed using Dunnett’s test. Significant differences were indicated by *p* < 0.05.

## 4. Conclusions

This study established a sequential separation process to fractionate three anti-inflammatory components from dried dulse without using organic solvents. The developed procedure consists of three key operations: (1) the weakening of heat-hardened cells, (2) the degradation of denatured proteins, and (3) the solubilization of chlorophylls. This procedure is a completely novel proposition in the algae-based industry and is clearly applicable to heat-dried algae containing difficult-to-extract components. Additionally, the three fractions obtained in this study showed inhibitory effects on the secretion of inflammatory cytokines induced by LPS, which were comparable to those of the dulse extract prior to the fractionation. The three fractions may possess distinct anti-inflammatory mechanisms of action, which could potentially broaden the applications of red algae as functional materials with anti-inflammatory properties.

## Figures and Tables

**Table 1:** Distribution ratios of sugar, phycobiliprotein-derived peptides (PPs), and chlorophyll-related compounds (Chls) contained in E1, E2, and E3.

Extract	Sugar	PPs	Chls
	% (mg/g Dried Thallus)	% (mg/g Dried Thallus)	% (mg/g Dried Thallus)
E1	91.7 (152.27 ± 1.47)	9.8 (19.50 ± 0.25)	0.8 (12.03 ± 0.42)
E2	7.6 (12.63 ± 0.41)	80.9 (160.83 ± 6.75)	3.6 (49.01 ± 3.53)
E3	0.7 (1.14 ± 0.05)	9.2 (18.45 ± 0.65)	95.5 (1287.95 ± 11.68)

E1, E2, and E3 are the final extracts in each step.

## Data Availability

The data presented in this study are available on request from the corresponding author.

## Supplementary Materials

The following supporting information can be downloaded at: https://www.mdpi.com/article/10.3390/md21050276/s1. Figure S1: Representative multiple reaction monitoring (MRM) chromatograms of E1 by sugar-component analysis for mono- and oligosaccharides. The E1 (shown in Figure 1) was analyzed using liquid chromatography (1260 Infinity II Prime Bio LC, Agilent Technologies, Ltd., Santa Clara, CA, USA) coupled with the tandem mass spectrometry (Ultivo Triple Quadrupole LC/MS/MS, Agilent Technologies, Ltd.). The separation column (InfinityLab Poroshell 120 HILIC-Z, 2.1×100 mm, 2.7 μm, Agilent Technologies) was maintained at 60 °C. Mobile-phase gradient elution was employed using the following conditions (A: 5 mM ammonium bicarbonate, pH 10.4, B: acetonitrile; 0–5 min: 95% B; 5–15 min: 95→80% B; 15–20 min: 80→70% B; 20–25 min: 70% B). The duration of the column equilibration was 12 min. The injection volume was 0.5 μL. The flow rate was 0.3 mL/min. Mass detection was performed using MRM in the negative-ion mode. The LC/MS/MS analysis revealed that E1 contained xylose, xylobiose, xylotriose (β-1,4 linkage), xylotriose (isomer, not β -1,4 linkage), and xylotetraose.
