# Supplementary material for: Development of a Sequential Fractionation-and-Recovery Method for Multiple Anti-Inflammatory Components Contained in the Dried Red Alga Dulse (Palmaria palmata)
Source: Mar Drugs. 2023 Apr 28;21(5):276. doi: 10.3390/md21050276 (PMC10221991; doi:10.3390/md21050276)
Supplement: Supplementary file 1 [file marinedrugs-21-00276-s001.zip › marinedrugs-2294655-supplementary.pdf]

Supplementary Materials:

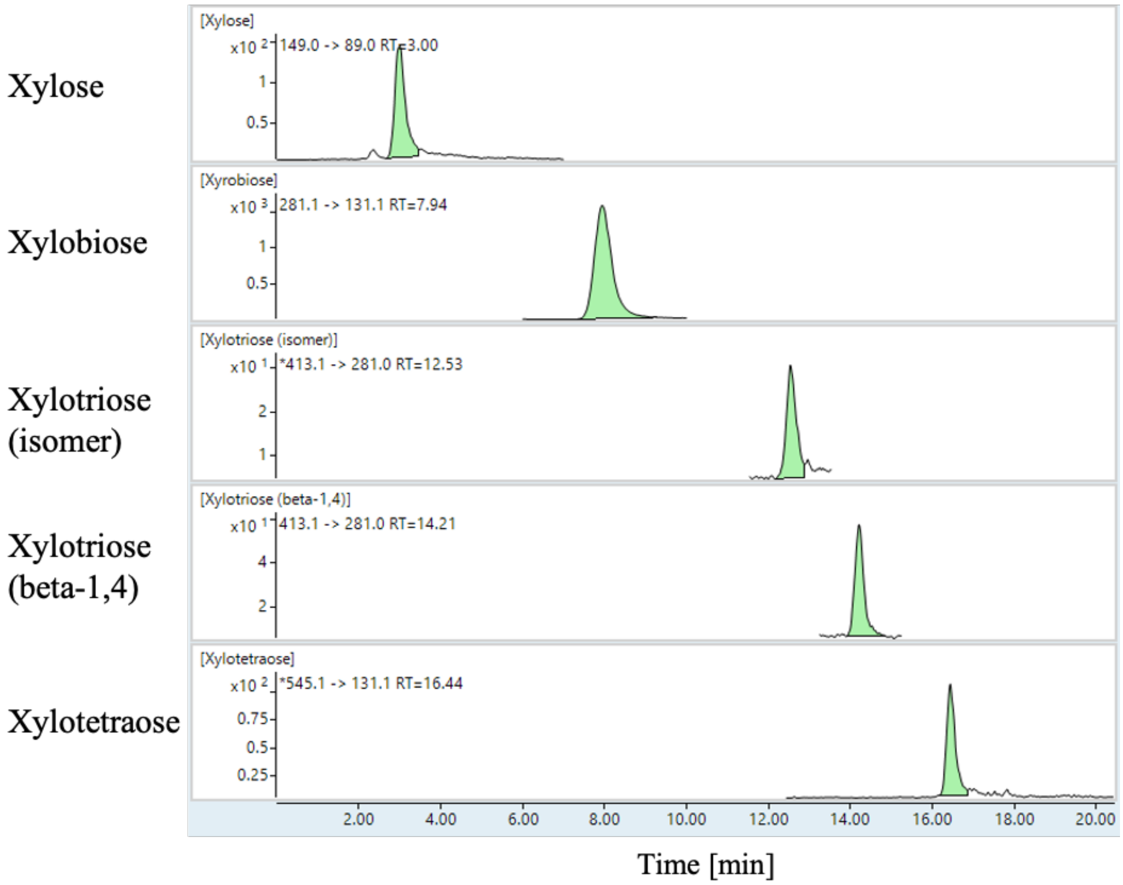

Figure S1: Representative multiple reaction monitoring (MRM) chromatograms of E1 by sugar component analysis for mono and oligosaccharides. E1 (shown in Figure 1) was analyzed using liquid chromatography (1260 Infinity II Prime Bio LC, Agilent Technologies, Ltd, Santa Clara, CA, USA) coupled with the tandem mass spectrometry (Ultivo Triple Quadrupole LC/MS/MS, Agilent Technologies, Ltd.). The separation column (InfinityLab Poroshell 120 HILIC-Z, 2.1 × 100 mm, 2.7 μm, Agilent Technologies) was maintained at 60° C. Mobile phase gradient elution was employed by the following conditions (A: 5 mM ammonium bicarbonate, pH 10.4, B: acetonitrile; 0–5 min: 95% B; 5–15 min: 95→80% B; 15–20 min: 80→70% B; 20–25 min: 70% B). The post time for the column equilibration was 12 min. The injection volume was 0.5 μL. The flow rate was 0.3 mL/min. Mass detection was performed using MRM in the negative ion mode. LC/MS/MS analy-sis revealed that E1 contained xylose, xylobiose, xylotriose (β-1,4 linkage), xylotriose (isomer, not β -1,4 linkage), and xylotetraose.
